# The complete chloroplast genome sequence of medicinal plant: *Peganum nigellastrum* (Zygophyllaceae)

**DOI:** 10.1080/23802359.2021.1950061

**Published:** 2021-07-19

**Authors:** Lei Zhang, Zhiheng Wang, Linxin Chang, Yuqing Wei

**Affiliations:** Key Laboratory of Ecological Protection of Agro-pastoral Ecotones in the Yellow River Basin, National Ethnic Affairs Commission of the People’s Republic of China, College of Biological Science & Engineering, North Minzu University, Yinchuan, PR China

**Keywords:** Zygophyllaceae, *Peganum nigellastrum*, chloroplast genome, phylogenetic analysis

## Abstract

*Peganum nigellastrum* is a medicinal plant. The total chloroplast (cp) genome length of *P. nigellastrum* is 160,066 bp, Containing a large single copy region of 88,275 bp, two inverted repeat regions of 26,486 bp and a small single copy region of 18,855 bp. The chloroplast genome contains 132 complete genes, including 87 protein-coding genes (87 PCGs), 8 ribosomal RNA genes (8 rRNAs), and 37 tRNA genes (37 tRNAs). The overall GC content of cp DNA is 37.5%, the corresponding values of the LSC, SSC, and IR regions are 35.6%, 31.4%, and 42.8%. Phylogenetic tree shows that *P. nigellastrum* has the closest relationship with *P. harmala*.

*Peganum nigellastrum* Bunge belongs to Zygophyllaceae, which is a medicinal plant in the kingdom planta used in folk medicines due to insecticidal activity (Rharrabe et al. 2007), inhibition of reproduction (Nath et al. 1993; Bibi 2017) and antimicrobial activity. The decoction of dried leaves and flowers are widely used to treat gastrointestinal, hypertension, cardiac, nervous system disorders and diabetes (Moloudizargari et al. 2013). However, the chloroplast (cp) genome of *P. nigellastrum* has not been reported. In this study, we assembled the complete chloroplast genome of *P. nigellastrum*, hoping to lay a foundation for further research.

Fresh leaves of *P. nigellastrum* were collected from Alxa Youqi (Alxa, Inner Mongolia, China; coordinates: 101.6757E, 39.2396 N) and dried with silica gel. The voucher specimen was stored in Sichuan University Herbarium with the number of QTPLJQCHNO0293002. Total genomic DNA was extracted with a modified CTAB method (Doyle and Doyle 1987) and a library with an average insert size of 350 bp was constructed. This library was sequenced on the Illumina NovaSeq 6000 system with 150 bp paired-end reads. We obtained 10 million high quality pair-end reads for *P. nigellastrum*, and after removing the adapters, the remaining reads were used to assemble the complete chloroplast genome by GetOrganelle pipeline v1.6.3a (Jin et al. 2020). The complete chloroplasts genome sequence of *P. harmala* was used as a reference. Plann v1.1 (Huang and Cronk 2015) and Geneious v11.0.3 (Kearse et al. 2012) were used to annotate the chloroplasts genome and correct the annotation.

The total plastome length of *P. nigellastrum* (MW970110) is 160,066 bp, with a typical quadripartite structural organization, consisting of a large single copy (LSC) region of 88,275 bp, two inverted repeat (IR) regions of 26,486 bp and a small single copy (SSC) region of 18,855 bp. The cp genome contains 132 complete genes, including 87 protein-coding genes (87 PCGs), 8 ribosomal RNA genes (8 rRNAs), and 37 tRNA genes (37 tRNAs). Most genes occur in a single copy, while 19 genes occur in double, including 4 rRNAs (4.5S, 5S, 16S, and 23S rRNA), 7 tRNAs (*trnA-UGC*, *trnE-UUC, trnL-CAA*, *trnM-CAU, trnN-GUU*, *trnR-ACG*, and *trnV-GAC*), and 8 PCGs (*rps7*, *rps19, ndhB*, *ycf2*, *ycf15*, *rpl2*, *rpl23* and *rpl32*). The overall GC content of cp DNA is 37.5%, the corresponding values of the LSC, SSC, and IR regions are 35.6%, 31.4%, and 42.8%.

In order to further clarify the phylogenetic position of *P. nigellastrum*, plastomes of 6 representative Zygophyllaceae species were obtained from NCBI to reconstruct the plastome phylogeny, with *Erodium absinthoides* as an outgroup. All the sequences were aligned by using MAFFT v.7.313 (Katoh and Standley 2013) and maximum likelihood phylogenetic analyses were conducted by using RAxML v.8.2.11 (Stamatakis 2014) under GTRCAT model with 500 bootstrap replicates. The phylogenetic tree shows that most the species of Zygophyllaceae were divided into two subclades (Figure 1). All *Nitraria* species clustered together, and all *Peganum* species clustered in another clade. while *P. nigellastrum has the closest relationship with P. harmala.*

**Figure 1.**
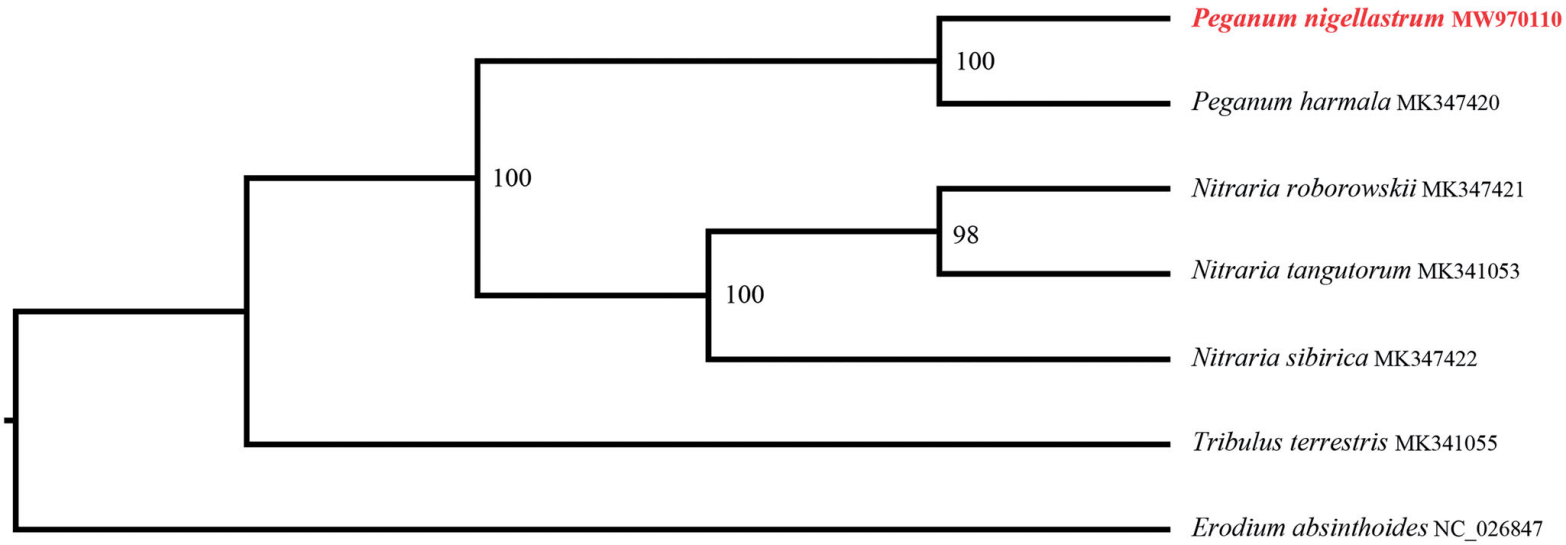
Maximum-likelihood phylogenetic tree for *Peganum nigellastrum* based on 7 species complete chloroplast genomes in family Zygophyllaceae.

## Data Availability

The data that support the findings of this study are openly available in GenBank of NCBI at https://www.ncbi.nlm.nih.gov, reference number MW970110. The associated BioProject, SRA, and Bio-Sample numbers are PRJNA672277, SRA: SRS8756899, and SAMN18837312, respectively.

## References

[CIT0001] Bibi F. 2017. Diversity of antagonistic bacteria isolated from medicinal plant *Peganum harmala* L. Saudi J Biol Sci. 24(6):1288–1293. 28855824 10.1016/j.sjbs.2015.09.021 PMC5562454

[CIT0002] Doyle JJ, Doyle JL. 1987. A rapid DNA isolation procedure for small quantities of fresh leaf tissue. Phytochem Bull. 19:11–15.

[CIT0003] Huang D, Cronk Q. 2015. Plann: a command-line application for anno-tating plastome sequences. Appl Plant Sci. 3(8):1500026. 10.3732/apps.1500026 PMC4542940 26312193

[CIT0004] Jin J-J, Yu W-B, Yang J-B, Song Y, dePamphilis CW, Yi T-S, Li D-Z. 2020. GetOrganelle: a fast and versatile toolkit for accurate *de novo* assembly of organelle genomes. Genome Biol. 21(1):241. 32912315 10.1186/s13059-020-02154-5 PMC7488116

[CIT0005] Katoh K, Standley D. 2013. MAFFT multiple sequence alignment software version 7: improvements in performance and usability. Mol Biol Evol. 30(4):772–780. 23329690 10.1093/molbev/mst010 PMC3603318

[CIT0006] Kearse M, Moir R, Wilson A, Stones-Havas S, Cheung M, Sturrock S, Buxton S, Cooper A, Markowitz S, Duran C, et al. 2012. Geneious basic: an integrated and extendable desktop software platform for the organization and analysis of sequence data. Bioinformatics. 28(12):1647–1649. 22543367 10.1093/bioinformatics/bts199 PMC3371832

[CIT0007] Moloudizargari M, Mikaili P, Aghajanshakeri S, Asghari MH, Shayegh J. 2013. Pharmacological and therapeutic effects of *Peganum harmala* and its main alkaloids. Pharmacogn Rev. 7(14):199–212. 24347928 10.4103/0973-7847.120524 PMC3841998

[CIT0008] Nath D, Sethi N, Srivastava R, et al. 1993. Studies on the teratogenic and antifertility of *Peganum harmala* L. Fitoterapia. 64:312–324.

[CIT0009] Rharrabe K, Bakrim A, Ghailani N, Sayah F. 2007. Bioinsecticidal effect of harmaline on *Plodia interpunctella* development (Lepidoptera Pyralidae). Pestic Biochem Physiol. 89(2):137–145.

[CIT0010] Stamatakis A. 2014. RAxML version 8: a tool for phylogenetic analysis and post-analysis of large phylogenies. Bioinformatics. 30(9):1312–1313. 24451623 10.1093/bioinformatics/btu033 PMC3998144

